# The Emulsifier Carboxymethylcellulose Induces More Aggressive Colitis in Humanized Mice with Inflammatory Bowel Disease Microbiota Than Polysorbate-80

**DOI:** 10.3390/nu13103565

**Published:** 2021-10-12

**Authors:** Esmat Rousta, Akihiko Oka, Bo Liu, Jeremy Herzog, Aadra P. Bhatt, Jeremy Wang, Mohammad B. Habibi Najafi, Ryan Balfour Sartor

**Affiliations:** 1 Center for Gastrointestinal Biology and Disease, Division of Gastroenterology and Hepatology, School of Medicine, University of North Carolina at Chapel Hill, 111 Mason Farm Road, Chapel Hill, NC 27599, USA; esmat267@email.unc.edu (E.R.); aoka@med.shimane-u.ac.jp (A.O.); bo_liu@med.unc.edu (B.L.); jeremy_herzog@med.unc.edu (J.H.); aadra_bhatt@med.unc.edu (A.P.B.); 2 Department of Food Science and Technology, Faculty of Agriculture, Ferdowsi University of Mashhad, Mashhad 9177948974, Iran; 3 Department of Internal Medicine II, Faculty of Medicine, Shimane University, 89-1, Enya, Izumo, Shimane 693-8501, Japan; 4 Center for Gastrointestinal Biology and Disease, School of Medicine, University of North Carolina at Chapel Hill, 111 Mason Farm Road, Chapel Hill, NC 27599, USA; jeremy_wang@med.unc.edu; 5 Department of Genetics, School of Medicine, University of North Carolina at Chapel Hill, 111 Mason Farm Road, Chapel Hill, NC 27599, USA; 6 Department of Microbiology and Immunology, School of Medicine, University of North Carolina at Chapel Hill, 111 Mason Farm Road, Chapel Hill, NC 27599, USA

**Keywords:** dietary emulsifiers, gut microbiota, experimental colitis, humanized mice, inflammatory bowel diseases, dietary triggers

## Abstract

Commonly used synthetic dietary emulsifiers, including carboxymethylcellulose (CMC) and polysorbate-80 (P80), promote intestinal inflammation. We compared abilities of CMC vs. P80 to potentiate colitis and impact human microbiota in an inflammatory environment using a novel colitis model of ex-germ-free (GF) IL10^−/−^ mice colonized by pooled fecal transplant from three patients with active inflammatory bowel diseases. After three days, mice received 1% CMC or P80 in drinking water or water alone for four weeks. Inflammation was quantified by serial fecal lipocalin 2 (Lcn-2) and after four weeks by blinded colonic histologic scores and colonic inflammatory cytokine gene expression. Microbiota profiles in cecal contents were determined by shotgun metagenomic sequencing. CMC treatment significantly increased fecal Lcn-2 levels compared to P80 and water treatment by one week and throughout the experiment. Likewise, CMC treatment increased histologic inflammatory scores and colonic inflammatory cytokine gene expression compared with P80 and water controls. The two emulsifiers differentially affected specific intestinal microbiota. CMC did not impact bacterial composition but significantly decreased Caudoviricetes (bacteriophages), while P80 exposure non-significantly increased the abundance of both Actinobacteria and Proteobacteria. Commonly used dietary emulsifiers have different abilities to induce colitis in humanized mice. CMC promotes more aggressive inflammation without changing bacterial composition.

## 1. Introduction

Resident intestinal bacteria promote mucosal homeostasis in normal individuals but induce inflammation in genetically susceptible hosts. Protection is mediated through microbial metabolites and induction/regulation of innate and adaptive homeostatic immune responses [1,2,3]. Conversely, bacterial subsets in combination with environmental factors activate effector TH1/TH17 immune responses that drive chronic, relapsing inflammation in genetically susceptible hosts [2,3]. This delicate balance between the composition and function of gut microbiota helps determine mucosal homeostasis vs. inflammation. An intact mucosal barrier prevents access of aggressive bacteria to effector immune cells [1,4]. Disruption of the protective mucosal barrier and dysregulated innate immune responses initiate the inflammatory response, but effector T cells are essential for disease progression and chronicity [5].

Dietary components are important modulators of the intestinal barrier and microbial metabolite production and can serve as triggers for flares of inflammatory bowel diseases (IBD). As an example, people living in urbanized parts of Asia consume greater amounts of Western food, compared to their rural counterparts. This is accompanied by an increased prevalence of IBD in urban areas [6]. Diet alters bacterial diversity and metabolism [7], but food additives present in Western diets can damage the mucosal barrier and potentiate inflammation [8,9]. Roberts et al. (2010) showed that dietary emulsifiers such as polysorbate-80 (P80) at the permitted level for food applications can increase *Escherichia coli* translocation across intestinal M cells and contribute to Crohn’s disease pathogenicity [10]. This finding stimulated many medical investigators and food scientists to pursue the possible adverse effects of these compounds in animal models [11,12,13,14,15].

Sodium carboxymethylcellulose (CMC) and P80 are the two most common emulsifiers used in the food industry [16]. Emulsifiers disperse immiscible phases to stabilize functional ingredients and flavorings in foods and beverages to extend their shelf life [17]. These compounds are primarily used in ice cream, cocoa drinks, dressing, bakery, and confectionery products at concentrations up to 1% [16]. They have been approved by the Food and Drug Administration (FDA) and affirmed as GRAS (generally recognized as safe) [18]. Despite FDA approval for food applications, CMC and P80 at low concentrations can induce damage of the intestinal mucosa and initiate metabolic syndrome and chronic inflammation. These compounds have no impact on germ-free (GF) mice. However, administration of CMC or P80, along with microbiota transplantation from emulsifier-treated mice, makes these mice more susceptible to inflammation and metabolic syndrome [11]. Consequently, these compounds appear to mediate their detrimental impacts by altering the composition and function of gut microbial subsets. Emulsifiers increase the motility and adherence of adherent/invasive *Escherichia coli* (AIEC) to intestinal epithelial cells and upregulate their virulence gene expression [19], enhance numbers of inflammatory bacteria in the intestinal tract and increase bacterial translocation across epithelia, thereby promoting inflammation and colon cancer in murine models [14]. Levine and colleagues demonstrated that a specialized diet that excludes presumed inflammatory foods common in the Western diet, including emulsifiers, maintains remission induced by enteral nutrition in Crohn’s disease patients [20]. 

In this study, we hypothesize that CMC and P80 have different abilities to induce intestinal inflammation in part due to their variable effects on gut microbiota. Since IL10^−/−^ mice have well-characterized time courses of T cell-mediated chronic colitis that is dependent on resident microbiota [21], the goal of our study is to assess the variable effects of CMC or P80 administration on humanized ex-GF IL10^−/−^ mice colonized with pooled feces from patients with active Crohn’s disease and ulcerative colitis. We determined the effects of emulsifiers on body weight, inflammatory markers, histologic inflammatory scores, and microbiota composition, abundance and functional pathways. Our results show that CMC more severely impacts experimental colitis in this humanized mouse model than does P80 and that these emulsifiers differentially affect microbial diversity and the relative abundance of specific intestinal bacterial and viral groups.

## 2. Materials and Methods

### 2.1. Animals

**Figure 1:**
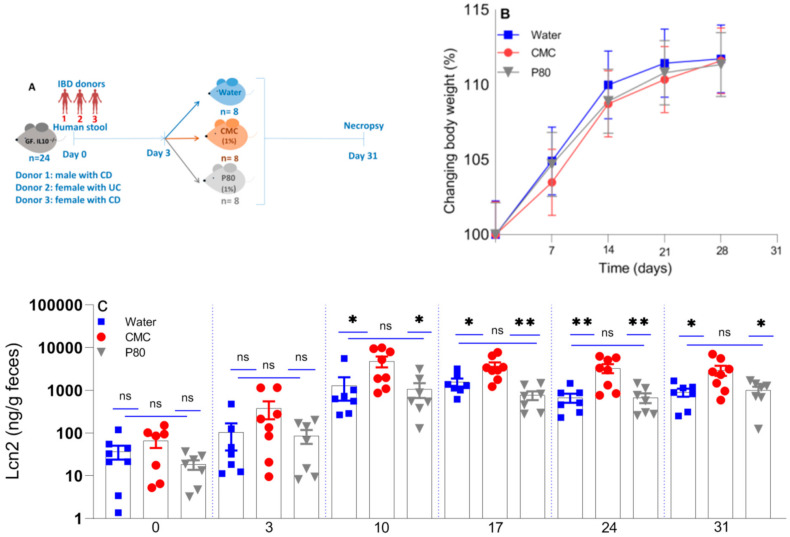
The impact of synthetic emulsifiers on body weight and an inflammatory fecal biomarker. In vivo experimental design (**A**), changes in body weight from baseline (**B**) and serial fecal Lcn-2 (**C**) in humanized IL10^−/−^ mice treated with CMC (1% *w*/*v*, red circle), P80 (1% *v*/*v*, gray triangle), and water (control, blue square). GF IL10^−/−^ mice were colonized with a pool of feces derived from three patients with active IBD and treated with water only, CMC (1% *w*/*v*) or P80 (1% *v*/*v*) in drinking water ad libitum for 31 days. The data are mean ± SEM of 7–8 individual mice. * *p* < 0.05; ** *p* < 0.01 by one-way ANOVA followed by Tukey multiple comparisons test. IBD; Inflammatory Bowel Diseases, CD; Crohn’s disease, UC; Ulcerative colitis, and ns; not significant.

GF male and female IL10^−/−^ mice on a 129SvEv background were provided by the National Gnotobiotic Rodent Resource Center at UNC-Chapel Hill, NC USA. The mice (7.5–10 weeks-old) were randomized into three groups of litter mates (N = 7–8 each) and housed in separate cages in a BSL2 isolator room in filtered top sterile cages under a 12-h light/dark cycle with ad libitum access to mouse chow (Selected Rodent 50 IF6F Auto) and water (Figure 1A). Mice were euthanized using carbon dioxide. All animal studies were approved by the Institutional Animal Care and Use Committee of the University of North Carolina (Protocol 18-266).

### 2.2. Materials

Sodium carboxymethylcellulose (average M*w*~250,000) and polysorbate-80 were purchased from Sigma-Aldrich Chemical Co. (St. Louis, MO, USA).

### 2.3. Colonization with Human Fecal Bacteria 

Stools were collected from three IBD patients with active disease, using an institutionally-approved protocol (IRB# 17-1528). Patient characteristics are described in Table 1. Freshly collected stools were transported to the laboratory on wet ice, then aseptically aliquoted in an anaerobic chamber and stored at −80 °C. Fecal slurries were prepared by vortexing 100 mg of each stool sample per milliliter of sterile, preconditioned phosphate-buffered saline for five minutes at room temperature. Solids were sedimented by centrifugation at 200 rpm for five minutes at 4 °C, and supernatants of individual samples were combined, and transferred to fresh tubes in anaerobic conditions. A total of 100 µL of the combined slurry was used to colonize GF mice by oral gavage.

### 2.4. Emulsifier Treatment

Three days after human fecal transplant, two groups of ex-GF mice received a solution containing 1% CMC or P80 (*w*/*v* and *v*/*v*, respectively) in drinking water ad libitum for four weeks. The third group (control) received only sterile reverse-osmosis (RO) water. The emulsifier solutions were prepared weekly in the bottles and autoclaved before use. The bottle contents were then transferred into the sterile containers assigned for mice under a laminar flow hood aseptically.

### 2.5. Lipocalin 2 Assay (Lcn-2)

Fresh fecal samples were collected weekly from the mice and frozen at −80 °C immediately to retain the integrity of the fecal microbiota. Lipocalin-2 (Lcn-2) values of fecal samples were measured by an ELISA kit (Lcn-2, R&D Systems, Minneapolis, MN, USA), according to the manufacturer’s protocols. To quantify the Lcn-2 values, 10–20 mg frozen fecal samples were dispersed in PBS containing 0.1% Tween 20 and incubated overnight at 4 °C. This slurry was briefly vortexed to obtain a homogenous fecal suspension and centrifuged for 10 min at 12,000 rpm and 4 °C. Lcn-2 concentrations were determined in the collected supernatants using the color reagents of hydrogen peroxide and tetramethylbenzidine at 450 and 570 nm by a microplate reader (Biotek Synergy HT, BioTek® Instruments, Inc., Winooski, VT, USA).

### 2.6. Colonic RNAs Extraction and qRT-PCR Analysis

At necropsy, distal colonic tissues were collected and placed in RNAlater (Qiagen, Hilden, Germany) at −20 °C. Total RNA was extracted using the RNeasy Mini Kit (Qiagen, Hilden, Germany) according to the manufacturer’s protocol. RNA concentration was measured using the NanoDrop system (Thermo Fisher Scientific, Madison, WI, USA). RNA was reverse transcribed to cDNA using the High Capacity cDNA Reverse Transcription Kit (Applied Biosystems, Foster City, CA, USA). Gene expression levels for murine interferon-gamma (IFN-γ), Interleukin 12p40 (IL-12p40), tumor necrosis factor alpha (TNF-α), and interleukin 1 beta (IL-1β) were quantified using iTaq Universal SYBR^®^ Green Supermix (Bio-Rad, Hercules, CA, USA) and specific mouse oligonucleotides (Table 2) via an RT-PCR system (Applied Biosystems). Gene expressions are presented as relative values using the ∆∆Ct approach with β-actin as an endogenous normalization control for primers.

### 2.7. Cytokine Measurement for Interferon Gamma Protein in Unstimulated Colonic Strip Cultures and Serum

As previously described and validated [22,23,24], fragment of each colon was placed in a Petri plate containing phosphate-buffered saline supplemented with 1% gentamicin under cold conditions, then transferred into a tube containing 20 mL PBS and shaken for a short time. The thoroughly washed tissues were then transferred into RPMI medium including 1% gentamicin and shaken for 30 min at 280 rpm at 22 °C. A portion of colon tissue of each sample (50 mg) was then placed into a well of a 24-well plate containing 1 mL of RPMI 1640 medium with 10% fetal bovine serum and 1% gentamicin (GIBCO) and incubated overnight in a CO_2_ incubator at 37 °C. The samples were centrifuged at 12,000 rpm for 10 min at 4 °C, and their supernatants were stored at −20 °C before IFN-γ quantification. Cardiac blood was collected at harvest. After clotting, serum was collected and stored at −80 °C until analysis.

IFN-γ protein concentration was determined by ELISA, according to the manufacturer’s protocols (#88-7314; Thermo Fisher Scientific, Invitrogen, Vienna, Austria). The levels of IFN-γ in cell cultures were calculated via a standard curve generated by recombinant murine IFN-γ and expressed as picograms IFN-γ per milligram of colonic tissue.

### 2.8. Myeloperoxidase Assay

Myeloperoxidase activity was quantified using an MPO colorimetric activity assay kit (#MAK068; Sigma-Aldrich, St. Louis, MO, USA), according to the manufacture’s instruction. Briefly, a portion of the cecum (50 mg) was homogenized in 4 volumes of MPO Assay Buffer, followed by centrifugation at 13,000× *g* for 10 min at 4 °C to remove insoluble material. Five µL of supernatant for each sample was added into a well of a 96 wells plate, and its volume was adjusted to a final volume of 50 µL with MPO Assay Buffer. The reaction was stopped after 60 min at room temperature away from light, and the absorbance at 412 nm was measured by a Clariostar Plate reader (BMG LABTECH, Cary, NC, USA). One unit of MPO activity was defined as the amount of enzyme that hydrolyzes the substrate and generates taurine chloramine to consume 1.0 µmole of trinitrobenzene sulphonic acid (TNB) per minute at 25 °C. The results were expressed as milliunit/mL.

### 2.9. Assessment of Histologic Inflammation

To determine the degree of inflammation, intestinal tissues fixed in 10% neutral buffered formalin for 24–48 h, then embedded in paraffin, sectioned (5 µm thickness), and stained with hematoxylin and eosin (H&E).The mucosal inflammation in the ileum, the cecum, proximal colon, distal colon, and rectum were blindly assessed according to a scoring system ranging from 0–4 for the degree of lamina propria and submucosal mononuclear cellular infiltration, crypt hyperplasia, goblet cell depletion, and architectural distortion. Histological scores are represented individually and as a sum of scores (maximum score = 12) for the entire large intestine, as previously validated [22,23,24].

### 2.10. Library Preparation and Shotgun Metagenomic DNA Sequencing

Whole bacterial DNA was extracted from frozen cecum contents (−80 °C) using AllPrep PowerViral DNA/RNA Kit (Qiagen, Hilden, NRW, Germany), according to the manufacturer’s instructions. The final DNA concentration and purity were determined using the NanoDrop system (Thermo Fisher Scientific, Madison, WI, USA).

The samples were prepared for DNA sequencing using Roche’s Kapa Hyper kit (Roche part# 07962363001) after a manual sonication of 45 s on the Covaris E220 (Covaris, Inc. Woburn, Massachusetts, USA). Target insert size post-sonication was ~400 base pairs. Libraries were constructed with the Hyper Library Preparation Kit from Kapa Biosystems (Roche Diagnostics, Indianapolis, Indiana, USA), followed by verification using Qubit (Thermo part# Q32854) and Tapestation D1000 Tape and reagents (Agilent Parts 5067-5582 and 5067-5583). Libraries were then pooled at equal molar concentrations. 

Samples were sequenced by the Illumina Novaseq 6000 (Illumina, Inc. San Diego, California, USA) using a version 1 chemistry 300 cycle kit on an SP flowcell (Illumina part# 20027465) for 2 × 150 cycles. The pool was diluted to 1.875 nM, denatured in 0.2 NaOH, neutralized with 400 nM Tris Buffer, and loaded on the flowcell for sequencing.

### 2.11. Microbial Analysis

The taxonomic composition of each sample was characterized from metagenomic sequence data using Kraken 2 and Bracken 2.6.0 software with default parameters. Differential abundance analyses were performed using ANCOM (Analysis of composition of microbiomes) with Holm–Bonferroni multiple-testing correction [25]. The Shannon index was calculated to measure alpha diversity (within-group diversity), and the pairwise difference in alpha diversity tested using a one-way ANOVA test. Data were visualized using principal coordinate analysis (PCoA) plots of the beta diversity (between groups diversity) computed by Bray–Curtis dissimilarity. The ANOSIM (analysis of dissimilarity) significance test was used to identify significance between the groups [26].

Metabolic pathway analysis was performed using HUMAnN 2.0 (https://huttenhower.sph.harvard.edu/humann2/, accessed on 7 October 2021) [27]. Differential abundance analysis of pathways was performed using ANOVA and ANCOM tests as described above for taxonomic composition.

### 2.12. Statistical Analysis of Non-Microbial Data

Statistical analysis for inflammatory parameters was conducted with Prism 8 software (GraphPad, San Diego, CA, USA). Significance between the groups was determined by analysis of variance (ANOVA), followed by an appropriate multiple comparisons test (*p* ˂ 0.05). All data are expressed as mean ± SEM.

## 3. Results

### 3.1. The Effect of CMC and P80 on Body Weight and Inflammatory Biomarkers

This study investigated the differential abilities of CMC and P80 to induce or exacerbate experimental colitis in 8–12 week-old humanized IL10^−/−^ 129SvEv mice. For this purpose, we colonized three groups of mice with pooled active IBD patients’ stools and evaluated evidence of progressive inflammation after continuous CMC or P80 administration in drinking water (1%) on Days 3–31 (Figure 1A). We recorded the body weight of each mouse weekly to monitor weight changes, as represented by individual percent change in body weight to account for different initial weights. As depicted in Figure 1B, the relatively young mice gradually gained weight over 28 days of the study, with no difference between groups at any time. The mice treated with CMC and P80 gained slightly less weight than the water controls, similar to results of Chassaing et al. (2015) for IL10^−/−^ mice treated with CMC or P80 solution (1%) for 90 days [11].

Lcn-2 is a sensitive fecal biomarker of intestinal inflammation [28,29]. As shown in Figure 1C, fecal Lcn-2 levels significantly increased from baseline after ten days of IBD fecal colonization in all groups, where values plateaued and remained elevated for the duration of the 31-day experiment. The mice that received CMC exhibited increased Lcn-2 levels compared with those that received P80 or water (control group) for the entire experiment. Ten days after human fecal transplant, seven days after beginning emulsifier treatment, mean Lcn-2 levels in CMC-treated mice were 4822 ng/g feces, 1068 ng/g for P80-treated mice, and 1299 ng/g for water-treated control mice (CMC vs. water and P80, *p* = 0.02). Chassaing et al. (2015) demonstrated that CMC (1% *w*/*v*) and P80 (1% *v*/*v*) each induced low-grade inflammation in wild-type mice and promoted robust colitis in IL10^−/−^ and TLR5^−/−^ C57Bl/6 mice after 12 weeks of administration [11]. In our model of more aggressive experimental colitis in ex-GF 129SvEv IL10^−/−^ mice humanized with pooled IBD fecal microbiota, CMC induced more active colitis than did P80 or water administration.

**Figure 2:**
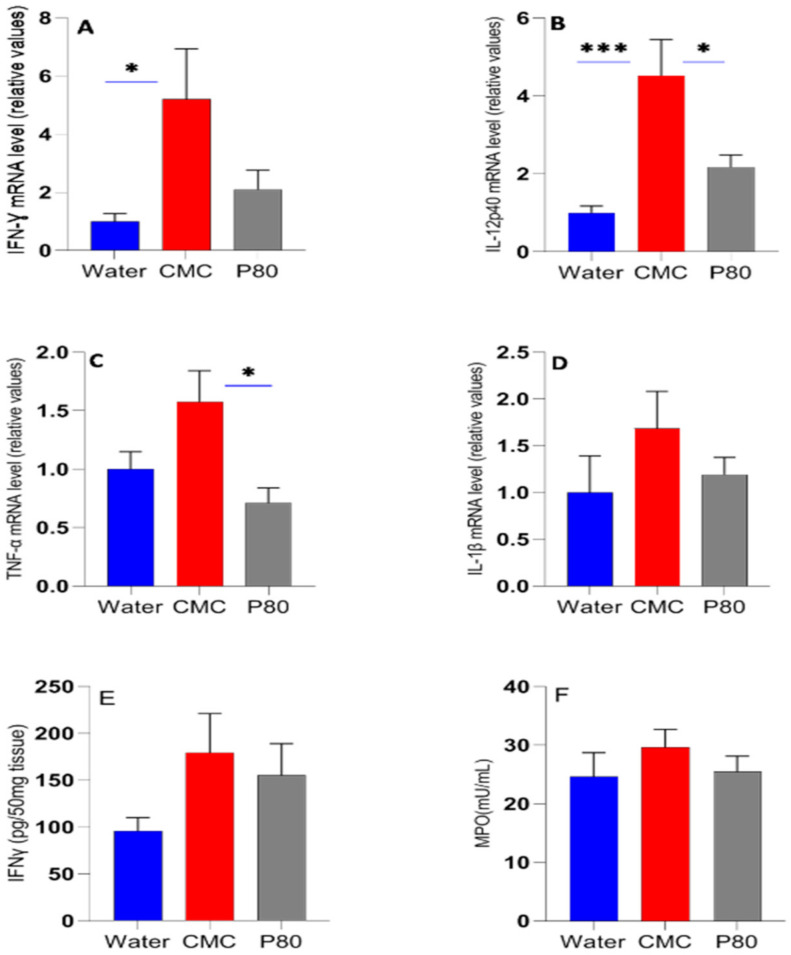
The distal colonic expression of IFN-γ, IL-12p40, TNF-α, and IL-1β mRNA (**A**–**D**), colonic IFN-γ concentration (**E**), and colonic MPO activity levels (**F**) in mice treated with CMC (1% *w*/*v*, red), P80 (1% *v*/*v*, gray), and water (control, blue) performed by qPCR. * *p* < 0.05; *** *p* < 0.001 by one-way ANOVA followed by Tukey multiple comparisons test.

Active IBD and experimental colitis are associated with the expression and secretion of a panel of inflammatory cytokines, including IL-1β, TNF-α, IL-6, IFN-γ and IL-17 in various cells such as immune cells and epithelial cells [3,23]. Accordingly, we measured the effect of CMC and P80 on the expression of representative colonic inflammatory cytokines by qRT-PCR analysis for gene expression. After 28 days of exposure, mice treated with CMC exhibited higher innate and adaptive inflammatory cytokine expression than P80 or water-treated mice (Figure 2A–F). For all cytokines measured, CMC exhibited highest levels, with significant increases over water controls for IFN-γ and IL-12p40 (Figure 2A,B) and nonsignificant differences for IL-6 (data not shown). The relative gene expression for IL-12p40 and TNF-α was higher for mice fed CMC compared to P80. IFN-γ, IL-12p40 and IL-1β levels were not significantly higher in P80-treated mice compared to water control. Interestingly, TNF levels were actually lower in the P80 group compared with water controls. CMC-treated mice exhibited a higher level of colonic IFN-γ protein than water treatment and to a lesser extent than P80, but with no statistically significant differences (Figure 2E). Serum IFN-γ and TNF-α levels were below detectible limits. MPO activity is a measure of neutrophil influx in colon tissue and indicates the degree of acute intestinal inflammation [14]. CMC-treated mice showed a higher, but nonsignificant colonic MPO activity compared with the water control and P80 (Figure 2F), indicating that CMC exposure increased neutrophil infiltration in the colon tissue.

### 3.2. CMC Enhanced Histologic Colonic Inflammation 

**Figure 3:**
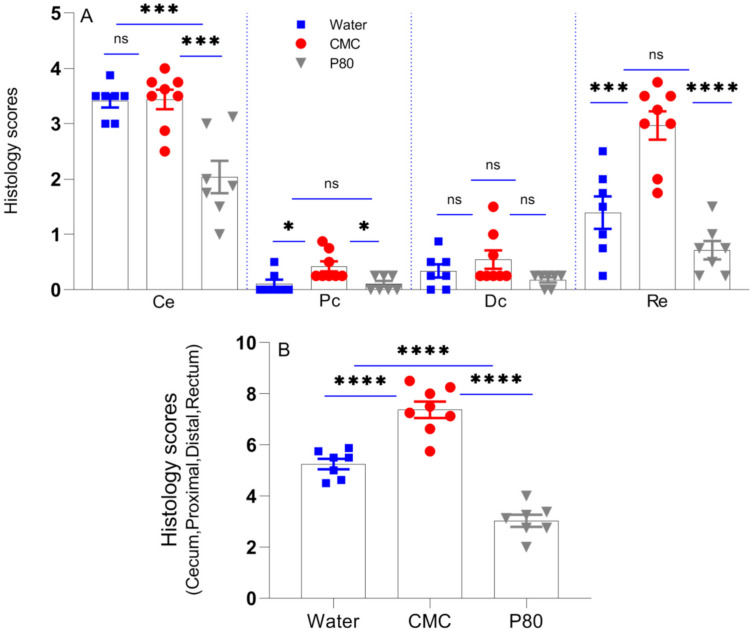
Blinded histological scores of the different sections of the colon (**A**) and overall scores (**B**) of humanized IL10^−/−^ mice exposed to CMC (1% *w*/*v*, red circle), P80 (1% *v*/*v*, gray triangle), and water (control, blue square). * *p* < 0.05; *** *p* < 0.001 and **** *p* < 0.0001 by one-way ANOVA followed by Tukey multiple comparisons test.

**Figure 4:**
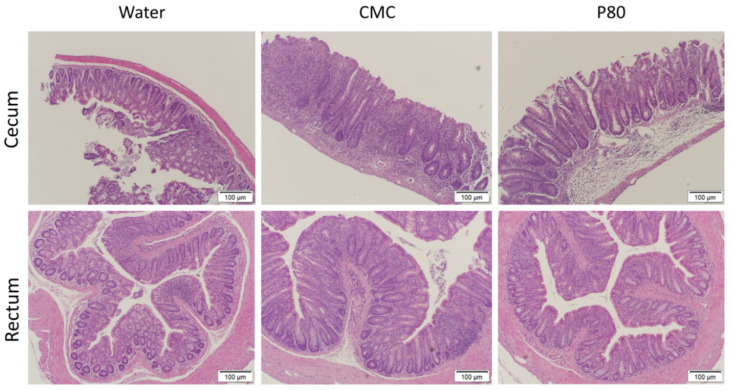
Representative histologic slides of the cecum and rectum stained by hematoxylin-eosin in humanized IL10^−/−^ mice after 31 days exposure to CMC (1% *w*/*v*), P80 (1% *v*/*v*), and water (control). Scale bar = 100 µm.

We performed blinded histological assessment of colitis on Day 31 as an independent assessment of the impact of CMC and P80 on colonic inflammation (Figure 3). Distinct regions of the colon were evaluated separately, and as in aggregate. Total histologic scores for the cecum, rectum and proximal colon were significantly higher in the CMC-treated group than other groups; however, the control group (water) exhibited higher histological scores than the P80 group (Figure 3A,B). These results were consistent with fecal Lcn-2 levels, and to a lesser extent, the tissue cytokine levels. This observation suggests that fecal lipocalin can better predict the activity of colitis than colonic cytokine gene expression, possibly because it represents the summation of inflammation throughout the colon. Colonic inflammation in the ex-GF IL10^−/−^ mice colonized with pooled IBD fecal microbiota was most prominent in the cecum and rectum, as we have noted in specific pathogen-free (SPF) IL10^−/−^ mice [21], and ex-GF IL10^−/−^ mice humanized with feces from a healthy human donor [4]. CMC significantly enhanced inflammation in the rectum and proximal colon compared with water alone, but had no potentiating effect in the cecum (Figure 3A). P80 treatment led to significantly lower histologic scores than water controls in the cecum, proximal colon and rectum. For all treatments, the ileum did not demonstrate any histologic abnormality. Histologic evidence of inflammation was moderate in humanized IL10^−/−^ mice treated with water, with crypt hyperplasia, loss of goblet cells and influx of mononuclear inflammatory cells into the colonic lamina propria, but not consistently in the submucosa (Figure 4). CMC exposure potentiated the crypt hyperplasia, increased the number of lamina propria mononuclear cells and led to occasional infiltration of the submucosa mononuclear cells and crypt abscesses. Overall, CMC exacerbated inflammation in IBD microbiota-colonized IL10^−/−^ mice and exhibited a more detrimental impact than P80, as measured by biochemical (fecal Lcn-2), immunologic (cytokine gene expression) and histologic parameters.

### 3.3. Differential Effects of CMC and P80 on Gut Microbiota Abundance and Composition

**Figure 5:**
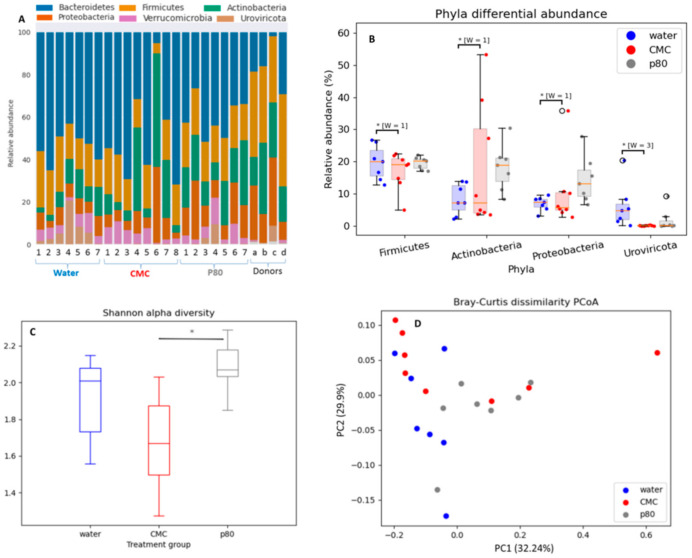
The effect of the CMC (1% *w*/*v*, red), P80 (1% *v*/*v*, gray), and water (control, blue) on cecal luminal microbiota composition (in percent) (**A**), the relative abundance of top four phyla (**B**), α-diversity in terms of Shannon index (**C**) and β-diversity in terms of Bray–Curtis dissimilarity (**D**) at the phyla levels analyzed by whole genome (shotgun metagenomics) sequencing in humanized IL10^−/−^ mice after 28 days exposure. Lowercase letters are IBD donors (a–c) and d is pooled feces of patients a, b, and c. Data are the means ± SEM (*n* = 7–8). * *p* < 0.05 by ANOVA with Holm–Bonferroni multiple-testing correction. * [W = X] indicates differential abundance by ANCOM with Holm–Bonferroni multiple-testing correction.

Previous studies show that synthetic emulsifiers commonly used in processed food are associated with intestinal inflammation and are dependent on altered gut microbiota composition [11]. We performed shotgun metagenomic analysis (whole genome sequencing) to elucidate the impact of CMC and P80 on cecal microbiota composition (Figure 5A–D). In our study, six phyla were predominant organisms in the gut, with descending order of abundance of Bacteroides, Firmicutes, Actinobacteria, Proteobacteria, Verrucomicrobia, and Uroviricota (Figure 5A). In this figure, the fecal microbiota composition of each IBD donor is indicated with lowercase letters (a–c) on the right side of Panel A, and Lane d is the microbiota composition of the pooled fecal slurry of patients prior to introduction into the mice. Certain phyla were affected differently by treatments. P80 showed a trend toward increased relative abundance of Actinobacteria and Proteobacteria, whereas CMC treatment significantly decreased the relative abundance of the viral phylum Uroviricota compared to water-treated control mice and P80 treatment (Figure 5B). CMC treatment did not significantly alter bacterial composition.

**Figure 6:**
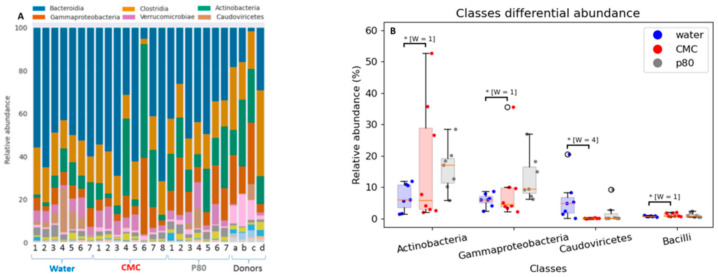
The effect of the CMC (1% *w*/*v*, red), P80 (1% *v*/*v*, gray), and water (control, blue) on cecal microbiota composition (in percent) (**A**) and the relative abundance of top four phyla (**B**), at the class levels analyzed via whole genome (shotgun metagenomics) sequencing in humanized IL10^−/−^ mice after 28 days exposure. Lowercase letters are IBD donors and d is a pooled fecal of a, b, and c. Data are the means ± SEM (*n* = 7–8). * [W = X] indicates differential abundance by ANCOM with Holm–Bonferroni multiple-testing correction.

The six most predominant microbial classes in descending order were Bacteroidia, Clostridia, Actinobacteria, Gammaproteobacteria, Verrucomicrobiae, and Caudoviricetes (Figure 6A). Four differentially abundant classes matched with the four phyla (Figure 6B) with P80 having a greater impact on microbiota composition alterations than did CMC. While P80 non-significantly increased the relative abundance of Actinobacteria and Gammaproteobacteria, CMC significantly decreased the relative abundance of the bacteriophage class Caudoviricetes compared with the control (water-treated) mice. Neither CMC nor P80 significantly altered individual species other than Bacteroides sp. CACC737 (P80 vs. water, w = 72) compared with water when corrected for multiple comparisons. Specifically, P80 did not increase either *E. coli*, *Klebsiella* or *Enterobacter genera*. *Ruminococcus gnavus* was increased in P80 vs. CMC (*p* = 00015). Again, the individual and pooled donor samples are provided in Figure 6A.

The Shannon alpha diversity index was significantly different between P80 and CMC-treated mice (*p* = 0.003), indicating increased microbial diversity after P80 treatment and decreased diversity after CMC. Unweighted principal coordinate analysis (PCoA) of beta diversity (Figure 5D) revealed that p80-treated mice had a significantly different fecal microbiota composition than the water- (*p* = 0.021) and CMC- (*p* = 0.049) treated mice after 28 days exposure. This finding was consistent with previous studies [19,30], which demonstrated that P80 affects the relative abundance of gut microbiota more directly than does CMC.

**Figure 7:**
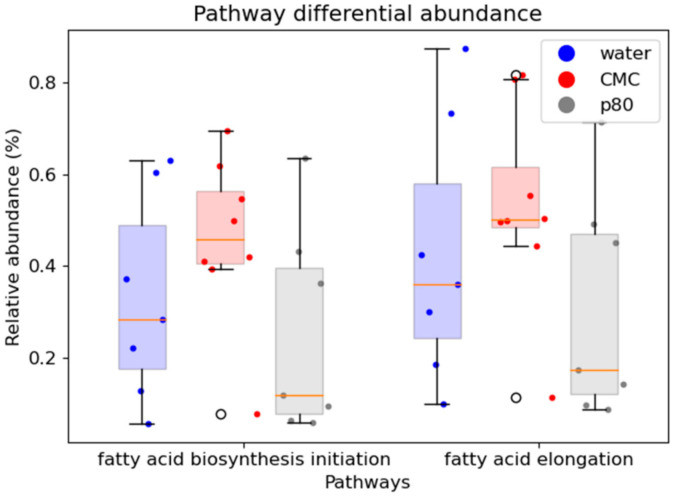
The effect of the CMC (1% *w*/*v*, red), P80 (1% *v*/*v*, gray), and water (control, blue) on select metabolic pathways. CMC illustrates a clear trend toward increased fatty acid biosynthesis initiation and elongation compared to P80 and water treatment. Medians and interquartile ranges are provided for each group.

An analysis of MetaCyc pathways using the metagenomic sequencing data failed to identify pathways that were significantly differently abundant between the groups. However, of potential importance to mechanisms of CMC pathogenicity, the mean value for the pathway for fatty acid biosynthesis in *E. coli* was increased 1.4-fold for CMC vs. water and 1.8-fold for CMC vs. P80, while fatty acid elongation mean was increased 1.7-fold for CMC compared with P80 (Figure 7).

While few significant microbial compositional and functional differences were found when corrected for multiple analyses, together these results indicate that while CMC does not induce major bacterial compositional differences, it affects microbial diversity and potentially alters bacterial functions to mediate its potentiation of experimental colitis in this novel humanized mouse model. 

## 4. Discussion

Our results indicate that CMC potentiates colitis in ex-GF IL10^−/−^ mice colonized with fecal microbiota from patients with active IBD to a greater degree than does P80, as measured by serial fecal lipocalin levels, blinded histologic scores, and colonic inflammatory cytokine expression. The greatest potentiation of histologic inflammation by CMC was evident in the colon, especially the rectum, with no differences in the highly inflamed cecum compared to water controls. Paradoxically, histologic cecal inflammation in P80-treated mice was less than water controls. These two commonly used dietary emulsifiers differentially affected the transferred IBD patient microbial compositions, with P80 selectively expanding Gammaproteobacteria and Actinobacteria to a minor degree, while CMC had no effect on bacterial profiles. In contrast, CMC decreased Uroviricota, driven by changes in the Caudoviricetes bacteriophage class. These clinically relevant results may have implications for the dietary management of IBD patients.

Our study has several unique features, most notably the use of highly susceptible GF 129SvEv IL10^−/−^ mice colonized as young adults with human complex microbiota by transplanting pooled feces from patients with active Crohn’s disease or ulcerative colitis. Mice colonized with human fecal microbiota is a helpful tool to study causal relationships between altered gut microbiota and human phenotypes [2,31]. However, to the best of our knowledge, we are the first to assess the impact of emulsifiers in such a model. In contrast, numerous studies have identified detrimental impacts of emulsifiers, particularly CMC and P80, in SPF and conventionally raised mice [11,14,19,30]. Together, these studies demonstrated that the emulsifiers exhibit pathophysiologic functions at doses below the levels reported for human dietary intake via processed foods [9]. At a 1% concentration, identical to the doses that we used, P80 and CMC were reported to have very similar abilities to induce colitis in conventional C57Bl/6 IL10^−/−^ mice [11]. However, 0.5% CMC (*w*/*v*) was the lowest concentration that resulted in low-grade inflammation (shortened colon, enlarged spleen) in the Toll-like receptor 5 (TLR5^−/−^) murine model and increased adiposity in wild-type (WT) mice, while P80 exhibited similar properties at a 0.1% (*v*/*v*) concentration. In these studies, the activity of inflammation depends on mice genotype, resident microbes, emulsifier type, and exposure period. GF mice are protected against CMC and P80, but WT mice colonized with SPF or low-complexity microbiota (Altered Schaedler Flora [ASF]) developed colonic inflammation induced by these emulsifiers [19]. Most studies have been conducted in SPF WT C57BL/6 mice. However, mice deficient in interleukin-10 (IL10^−/−^) or TLR5^−/−^ mice are more responsive to emulsifier exposure than are SPF and ASF WT mice. Colitis in our model of experimental colitis in ex-GF 129SvEv IL10^−/−^ mice humanized with pooled IBD fecal microbiota was more aggressive and occurred more rapidly than those of previous studies with SPF or conventionally raised C57BL/6 background mice. Chassaing et al. (2015) demonstrated that 1% CMC and P80 induced low-grade inflammation in WT C57BL/6 mice and promoted robust colitis in IL10^−/−^ C57BL/6 mice that reached peak intensity after 8 weeks of emulsifier administration [11]. SPF WT C57BL/6 mice housed under Helicobacter positive conditions and treated with DSS and azoxymethane showed similar colitis markers following exposure to 1% CMC or P80 [14]. In our humanized model, Lcn-2 concentration, a sensitive fecal biomarker of intestinal inflammation, reached maximal levels after 10 days exposure to these emulsifiers. We believe that our model of ex-GF genetically susceptible mice colonized with IBD patient microbiota enhances the clinical relevance of our observations. 

We observed in our model that CMC exhibited greater inflammatory potential than P80 by inducing more active colitis than did P80. In contrast, as noted above, Chassaing et al. (2015) and Viennois et al. (2018) reported roughly similar degrees of colitis with P80 and CMC (1%) in IL10^−/−^ C57BL/6 mice and SPF C57BL/6 mice treated with DSS and azoxymethane, respectively [11,14]. Active IBD and experimental colitis are associated with the expression and secretion of many pro-inflammatory cytokines, including IL-1β, TNF-α, IL-6, IFN-γ and IL-17 by innate and adaptive immune cells [3,23]. We confirmed our histologic observations by showing that CMC-treated mice expressed higher inflammatory cytokines than P80-treated mice. Together these fecal biomarker, histologic and immunologic results consistently showed that CMC induced more active colonic inflammation than P80 and water. The P80 treatment group showed statistically lower inflammatory readouts than water controls only in the total and cecal histologic scores, which were not confirmed by biochemical biomarkers (lipocalin and MPO) or cytokine gene expression or protein levels of IFN-γ but the P80 group show consistently lower values than CMC treatment by all readouts. It should be noted that regional histologic scores varied between the different colonic regions, while fecal lipocalin represents a composite of all intestinal regions, but is most heavily influenced by the distal colon and rectum. The cytokine measurements were made using distal colonic tissues and MPO from cecal tissues, perhaps explaining differences in the various readouts of inflammation.

The mechanisms that mediate the differential abilities of these two emulsifiers to induce variable degrees of colonic inflammation are not entirely clear. Histologic evidence of colonic inflammation in the ex-GF IL10^−/−^ mice colonized with pooled IBD fecal microbiota was most prominent in the cecum and rectum, as noted in SPF 129SvEv IL10^−/−^ mice [21] and ex- GF IL10^−/−^ mice humanized with feces from a healthy human donor [4]. Of potential importance, we observed no significant differences in histologic scores in the cecum and distal colon with CMC compared to the water controls. In contrast, P80-exposed mice developed less inflammation than water controls in the cecum and rectum. The reason for these differences in site of action is unknown, but could be related to differential effects of these emulsifiers on regional microbiota profiles. *Gammaproteobacteria* were selectively increased in the cecal lumen by P80, but not CMC. *Gammaproteobacteria* are reproducibly increased in patients with active IBD [32,33], and AIEC induce cecal-predominant colitis in monoassociated gnotobiotic 129 SvEv IL10^−/−^ [19]. These results appear paradoxical, since P80 treatment decreased colitis in the cecum relative to both water controls and CMC administration, despite the increased number of *Gammaproteobacteria*. Possible explanations are that *Klebsiella pneumonia, E coli* and *Enterobacter species*, which are resident Enterobacteriaceae species that are expanded in IBD patients and have documented abilities to induce colitis in gnotobiotic mice, were not increased in P80– treated mice [3,22,32,34]. Likewise, *R. gnavus* was increased by P80 compared with CMC treatment. *R. gnavus* can be increased in Crohn’s disease, but is a common resident bacterial species that is found in 90% of healthy humans and only one of two primary clades are selectively associated with Crohn’s disease [35]. However, in addition, P80 also increased the relative abundance of Actinobacteria in the cecal contents compared to water controls, while CMC-treated mice exhibited a broad range of Actinobacteria concentrations. The *Actinobacteria phylum* and class contain a beneficial commensal genus (*Bifidobacteria*). Multiple *Bifidobacteria* species have validated protective effects and are clinically used as probiotics. In this regard, *Bifidobacterium* species can protect the host against pathogens by producing bacteriocins, lowering the luminal pH, and preventing pathogens from adhering to the intestinal mucosa [36]. In addition, *Bifidobacterium longum* subsp. longum BB536 can abrogate inflammatory conditions in ulcerative colitis patients via improving mucosal barrier function by upregulating tight junction molecules [37]. These results suggest that P80 may stimulate a more protective microbiota composition than does CMC in our model. The concept of P80 inducing more protective microbiota is supported by higher bacterial diversity and richness in P80-treated mice compared with CMC treatment [38]. Similarly, we showed higher Shannon-α diversity of the microbiome in P80 vs. CMC-treated mice, and a trend toward higher diversity in P80-treated mice relative to water controls. Decreased microbial diversity following CMC and increased diversity following P80 treatment is consistent with the well-known reciprocal relationship between microbial diversity and inflammation [3,31,32]. Interestingly, CMC treatment decreased the relative abundance of the Uroviricota phylum and Caudoviricetes class compared to water-treated mice (control). This observation was contrary to the observed expansion of *Caudovirales bacteriophages* in IBD patients [39], suggesting that CMC may adversely affect phage-bacterial interactions in the gut. The bacterial specificity of these depleted bacteriophages is unknown, but we speculate that decreased killing of resident intestinal pathobionts might permit expansion of disease-inducing bacterial strains such as AIEC that may not be reflected in our metagenomic profiling.

Despite its relative inability to alter bacterial composition, CMC has been reported to selectively alter *E. coli* function as a proposed mechanism of inducing colitis. CMC rapidly altered the bacterial transcriptome in an ex vivo culture model to express virulence factors such as flagella and facilitate bacterial adherence to epithelial cells and increase their penetration of the mucus layer [30], with direct effects on the AIEC strain LF82 in vitro [19]. In contrast, P80 gradually induces flagella-related gene expression and more directly alters microbiome composition [29]. It is possible that we may observe a more aggressive course of colitis with P80 administration if we expanded our study beyond four weeks. Consistent with altered bacterial functions, in our metagenomic analysis CMC exposure was associated with expansion of the fatty acid biosynthesis pathway for *E. coli* and the fatty acid elongation pathway, with P80 treatment having the opposite effect.

Our findings have potential clinical implications. Exclusion diets have been used to induce remission in patients with refractory Crohn’s disease and prevent relapse after enteral nutrition induction of remission [20,40]. However, this diet requires a firm commitment from the patients to avoid all other food intake. It is possible that a low CMC diet might be an alternative exclusion diet for people with Crohn’s disease owing to its higher palatability and ease of use [41]. 

## 5. Conclusions

This study shows that CMC has detrimental effects on colonic immunologic and histologic parameters in ex-GF IL10^−/−^ mice colonized with pooled IBD fecal transplants, inducing greater inflammation in this model than does P80. These commonly used synthetic emulsifiers appear to act via different mechanisms, with P80 directly stimulating both aggressive and protective bacterial subsets, while CMC has no effect on bacterial composition, but selectively decreases microbial diversity and bacteriophages. 

## Figures and Tables

**Table 1:** IBD stool donor patient characteristics.

IBD Donor ^1^	Race	Sex	Disease
1	Caucasian	M	CD
2	Caucasian	F	UC
3	African-American	F	CD

^1^ All donors had active disease. IBD; inflammatory bowel diseases, CD; Crohn’s disease, UC; ulcerative colitis.

**Table 2:** Primer Sequences.

Gene	Type	Sequence (5’-3’)
β Actin	Forward Reverse	AGCCATGTACGTAGCCATCCAG TGGCGTGAGGGAGAGCATAG
IFN-γ	Forward Reverse	CTTCCTCATGGCTGTTTCTGG ACGCTTATGTTGTTGCTGATGG
IL-12p40	Forward Reverse	CGCAAGAAAGAAAAGATGAAGGAG TTGCATTGGACTTCGGTAGATG
TNF-α	Forward Reverse	ACCCTCACACACATCAGATCATCTTCTC TGAGATCCATGCCGTTGG
IL-1β	Forward Reverse	GTGGACCTTCCAGGATGAGG CGGAGCCTGTAGTGCAGTTG

## Data Availability

The relevant data are available from the authors upon reasonable request. We will deposit the metagenomic sequencing data on a publicly available web site such as GENBANK prior to publication and provide this link for unrestricted public access.
